# Improved Outcomes with Early Functional Rehabilitation After Reverse Shoulder Arthroplasty for Proximal Humerus Fractures in Older Patients

**DOI:** 10.3390/jcm15093284

**Published:** 2026-04-25

**Authors:** Michael Kimmeyer, Simon Keller, Christian Gerhardt, Verena Rentschler, Stefanie Kaiser, Johannes Kirsch, Michael Hackl, Lars-Johannes Lehmann

**Affiliations:** 1 Department of Orthopaedic and Trauma Surgery, University Medical Centre Mannheim, Medical Faculty Mannheim, University of Heidelberg, Theodor-Kutzer-Ufer 1-3, 68167 Mannheim, Germany; 2 Medical Faculty Mannheim, University of Heidelberg, Theodor-Kutzer-Ufer 1-3, 68167 Mannheim, Germany; 3 Department of Traumatology, Hand Surgery and Sports Medicine, ViDia Clinics Karlsruhe, Steinhaeusserstr. 18, 76135 Karlsruhe, Germany; 4 Department of Diagnostic and Interventional Radiology, ViDia Clinics Karlsruhe, Steinhaeusserstr. 18, 76135 Karlsruhe, Germany

**Keywords:** RSA, geriatric, tuberosity refixation, rehabilitation

## Abstract

**Background and Objective:** This study aimed to evaluate the impact of early functional rehabilitation on clinical outcomes and tuberosity healing in older patients undergoing reverse shoulder arthroplasty for proximal humeral fractures. We hypothesized that early functional rehabilitation would not compromise tuberosity healing and would result in comparable or improved outcomes versus postoperative immobilization. **Methods:** This retrospective matched-pair analysis included patients aged 70 years or older who underwent reverse shoulder arthroplasty for proximal humeral fractures, with 12 to 24 months of follow-up. Group allocation was time-based: earlier patients received immobilization and later patients underwent early rehabilitation. Matching was based on sex, age, body mass index, fracture classification (Neer), and glenosphere size. Outcomes included patient-reported scores, range of motion, and radiographic assessment of tuberosity healing using standardized imaging. **Results:** Forty patients (20 per group) with a mean age of 80.7 years and a mean follow-up of 16.1 months were included. The early rehabilitation group demonstrated significantly higher Constant scores (*p* = 0.044), age- and sex-adjusted Constant scores (*p* = 0.033), and greater active external rotation (*p* = 0.002). Anatomical tuberosity healing was seen in 28 of 40 patients (70%). Greater tuberosity healing occurred in 75% and lesser tuberosity healing in 85% of patients with available axial imaging. One deep infection occurred in the early rehabilitation group and was successfully managed. **Conclusions:** Early functional rehabilitation after reverse shoulder arthroplasty in older adults with proximal humerus fractures improved functional outcomes without compromising tuberosity healing.

## 1. Introduction

Reverse shoulder arthroplasty (RSA) has emerged as a well-established surgical option for treating complex proximal humeral fractures (PHFs), particularly in geriatric populations [1]. Advances in prosthesis design have contributed to improved outcomes in fracture healing and shoulder function recovery [2]. However, despite its increasing use for PHFs, there is currently no consensus on the optimal postoperative rehabilitation strategy [3]. Traditionally, the shoulder is immobilized in an abduction brace for several weeks to promote fracture healing and secure tuberosity refixation [3]. This restrictive approach prioritizes biological healing, yet may delay functional recovery and independence, especially in frail older patients with multiple comorbidities or limited mobility who require walking aids such as rollators, walking frames, or forearm crutches [4]. Recent studies suggest that early functional rehabilitation (EFR) may improve range of motion and patient-reported outcomes following RSA for PHFs [5]. However, concerns remain regarding the impact of early mobilization on anatomical healing of the greater tuberosity (GT), as some studies report lower healing rates and increased malunions in this context [5]. Although tuberosity healing has been associated with improved postoperative shoulder function [6], the relationship is likely influenced by multiple factors, and causality has not been firmly established. Thus, the potential trade-off between early mobility and anatomical healing remains an unresolved clinical question.

The objective of this study was to compare EFR with a postoperative immobilization protocol (non-EFR) in older patients undergoing RSA for PHFs. Specifically, we aimed to evaluate the effect of EFR on clinical outcomes and tuberosity healing. We hypothesized that EFR would not compromise tuberosity healing and would result in comparable or improved clinical and patient-reported outcomes compared to non-EFR.

## 2. Methods

### 2.1. Patient Selection

Patients undergoing RSA were systematically followed and recorded in the national shoulder prosthesis registry after providing written informed consent. Ethical approval was obtained from the Ethics Committee II of Heidelberg University (#2024-894).

All consecutive RSAs for acute PHFs performed between 1 June 2022, and 1 December 2023 were screened. All surgeries were performed by the same team of orthopedic and traumatology surgeons, specialized in shoulder surgery, at a shoulder and elbow surgery center certified by the national shoulder and elbow society. Inclusion criteria were age 70 years or older, surgery within 14 days post-trauma, and a minimum of 12 months and a maximum of 24 months of complete clinical and radiographic follow-up. Patients without follow-up were excluded.

### 2.2. Indication for Surgery, Implant Design, and Surgical Technique

RSA was indicated for PHFs with significant displacement or comminution, particularly in high-risk patients with advanced age, comorbidities, or fractures unsuitable for osteosynthesis or conservative treatment. The goal was to restore shoulder function and independence.

Regional anesthesia was used in most cases. Surgeries were performed in the beach-chair position via a deltopectoral approach. Tuberosities were identified and osteotomized when necessary. After humeral head resection and glenoid preparation, the prosthetic components were implanted. A single implant design was used in all cases (Univers Reverse, Arthrex, Naples, FL, USA) with a 135° humeral inclination and a lateralized glenosphere (36 + 4 mm or 39 + 4 mm), selected according to anatomical fit. Components were implanted at 20° retroversion using a cementless press-fit technique. The choice of baseplate fixation (central screw vs. central post) was made intraoperatively based on bone quality, with central screw fixation preferentially used in cases of osteoporosis, poor bone quality, or thin glenoid bone. In most cases, the modular glenoid system was used, whereas in a few cases its predecessor, the universal glenoid, was implanted.

**Figure 1:**
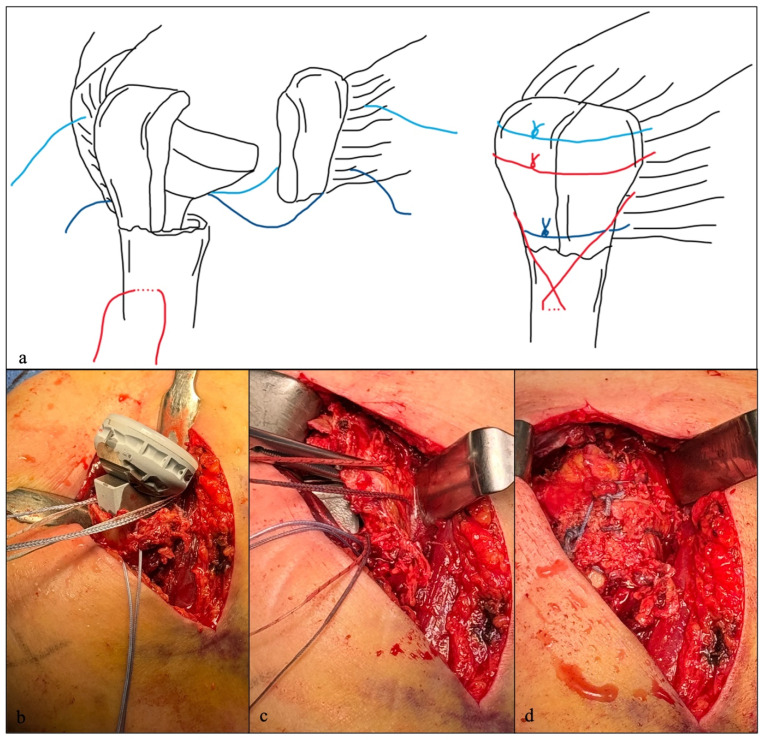
Reverse shoulder arthroplasty (RSA) for a proximal humerus fracture. (**a**): Schematic illustration of the tuberosity refixation technique with one vertical and two horizontal sutures. (**b**–**d**): Surgical technique with implantation of a 135° RSA and refixation of the tuberosities.

Tuberosity fixation was performed using a standardized cerclage technique with both horizontal and vertical non-resorbable sutures (FiberTape, Arthrex, Naples, FL, USA) (Figure 1) [7]. Sutures were passed transosseously through the proximal humerus and anchored to the prosthesis cup (Univers Reverse Suture Cup, Arthrex, Naples, FL, USA). After tuberosity reduction and temporary fixation, horizontal cerclages were tied first, followed by vertical sutures, aiming for complete osseous and soft tissue coverage to ensure stability and mobility.

### 2.3. Rehabilitation Protocols

Two rehabilitation protocols were evaluated in this study. Initially, the non-EFR protocol was the standard approach. In the second half of the study period, the EFR protocol was increasingly implemented. Both rehabilitation protocols are described in Appendix A and Appendix B.

In the EFR protocol, a shoulder sling (Tricodur Gilchrist Plus, BSN medical, Hamburg, Germany) was worn optionally for a few days, depending on pain. Pain-free passive and active-assisted movements, including external rotation, were initiated on postoperative day 1. Physiotherapy was continued multiple times per week following discharge.

In the non-EFR group, immobilization for three weeks in a shoulder abduction brace (Ultrasling Pro, Enovis, Carlsbad, CA, USA) set at 15° abduction was standard. Passive motion was limited to 60° of abduction/flexion; no active motion or external rotation beyond 0° was permitted during the first six weeks. Afterward, unrestricted active mobilization was initiated. Physiotherapy was conducted regularly throughout.

### 2.4. Variables

Patient characteristics and treatment characteristics were collected. Patient-reported outcome measures (PROMs) included subjective shoulder value (SSV) [8], visual analogue scale (VAS), Constant score (CS) [9], age- and gender-adjusted Constant score (ACS) [10], and the Disabilities of the Arm, Shoulder, and Hand score (DASH) [11]. Clinical evaluation included active and passive range of motion (ROM) (abduction, flexion, internal/external rotation) and isometric abduction strength at 90° in the scapular plane. Revision was defined as any unplanned surgery involving the ipsilateral glenohumeral joint. Complications were defined as adverse events related to RSA negatively affecting outcomes.

### 2.5. Radiological Evaluation

Preoperative radiographs were classified according to Neer [12] and AO (Arbeitsgemeinschaft für Osteosynthesefragen) [13]. Postoperative evaluation included reduction in tuberosities. At follow-up, imaging consisted of true anteroposterior (AP), outlet (Y), and, if available, axial views. GT healing was assessed primarily in true AP and Y views and if available in axial views. Lesser tuberosity (LT) healing was assessed in axial views. Tuberosity outcomes were categorized as healed, migrated, malunited, or resorbed. Healing, migration, and malunion were assessed in a binary manner, whereas resorption was further classified as none, partial, or complete. Other findings included periarticular ossifications and scapular notching [14]. Radiographs were assessed and evaluated by two authors (M.K., J.K.) using a standardized protocol and then reviewed in consensus with the senior author (L.L.) to reach consensus.

### 2.6. Statistical Analysis

Patients were grouped based on the rehabilitation protocol. EFR patients were matched 1:1 with non-EFR patients using propensity scores based on sex, age, BMI, fracture type (Neer classification), and glenosphere size. Matching adequacy was verified by evaluating mean and maximum distance scores. Statistical analysis was performed using SPSS^®^ version 28.0 (IBM^®^, Armonk, NY, USA). Continuous variables with normal distribution were expressed as mean ± standard deviation (SD), continuous variables with non-normal distribution were expressed as median and interquartile range (IQR), and categorical data were reported as percentages. Data normality was assessed with the Shapiro–Wilk test. Independent *t*-tests or Mann–Whitney U tests were used depending on distribution; Pearson’s chi-square test was used for categorical variables. Statistical significance was set at *p* < 0.05. Inter-rater reliability of radiographic analysis between a shoulder trained surgeon (M.K.) and a senior radiologist (J.K.). was evaluated using Cohen’s kappa (κ). Values were interpreted as follows: <0.20 slight, 0.21–0.40 fair, 0.41–0.60 moderate, 0.61–0.80 substantial, and 0.81–1.00 almost perfect agreement.

## 3. Results

**Figure 2:**
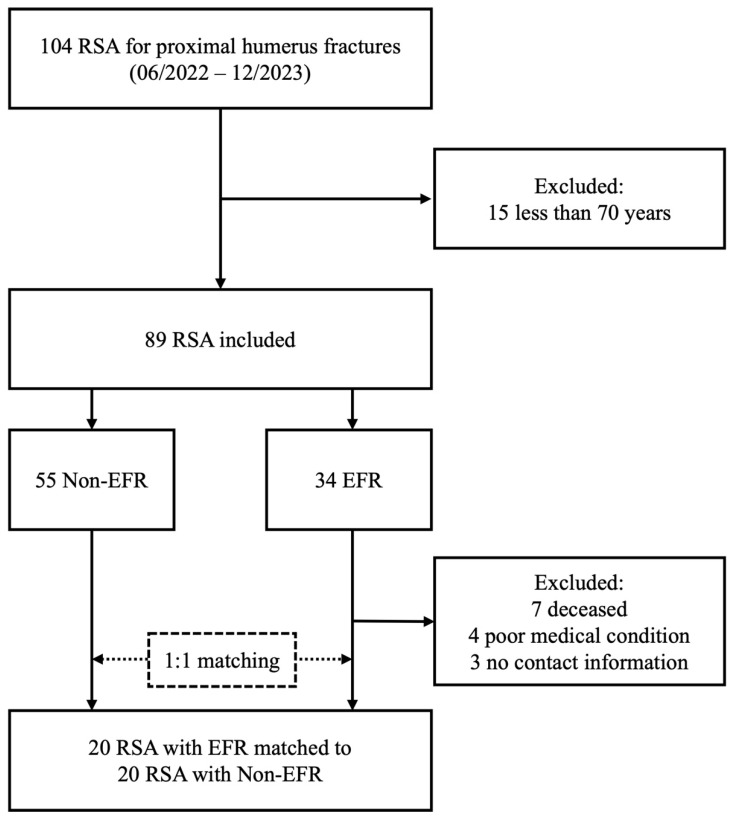
Study inclusion flowchart (RSA: reverse shoulder arthroplasty, EFR: early functional rehabilitation, non-EFR: non-early functional rehabilitation).

A total of 89 patients who underwent RSA for PHF were identified (Figure 2). Among 34 RSAs in the EFR group, 20 patients had complete functional and radiographic follow-up and were included in the analysis. These 20 EFR cases were matched to 20 RSAs from the non-EFR group. The mean propensity score distance between matched pairs was 0.09, with a maximum distance of 0.18.

### 3.1. Study Cohort

The mean age at surgery was 80.7 ± 6.5 years, and the majority of patients were female (36/40, 90%). The median follow-up duration was 15 months (IQR 6.8). The ASA score was significantly higher in the EFR group (*p* = 0.046). Outpatient nursing services was required in 16 patients (40%) for assistance with daily activities and hygiene. Fracture pattern differed significantly between the groups according to the Neer classification (*p* = 0.038), but not according to the AO classification (*p* = 0.432). Other baseline characteristics were similar between the groups (Table 1). In most cases, a 36 + 4 mm glenosphere was used (32/40, 80%). Anatomical reduction and correct prosthesis placement were achieved in all RSAs. Treatment characteristics were similar across the groups (Table 2).

### 3.2. Clinical Outcomes

At the final follow-up, the median VAS pain score was 0.0 (IQR 3.0), with no significant differences between groups. The mean SSV did not differ significantly between the EFR and non-EFR groups (78.0 ± 13.6 vs. 70.8 ± 14.4; *p* = 0.111). The mean CS and mean ACS were 60.7 ± 15.0 and 68.9 ± 15.9, respectively. The CS was significantly higher in the EFR group compared to the non-EFR group (65.45 ± 12.48 vs. 55.95 ± 15.26; *p* = 0.044). The EFR group demonstrated significantly greater active external rotation than the non-EFR group (35.35 ± 13.35 vs. 20.65 ± 15.06; *p* = 0.002). Full clinical and PROM results are presented in Table 3.

### 3.3. Radiological Outcomes

Radiographs were available for all patients. AP and Y views were available in all cases; axial views were available in 65%, allowing LT healing assessment in 13 patients per group. Interrater reliability was substantial for general tuberosity healing, GT healing, scapular notching, and ossifications (κ = 0.650–0.762) and moderate for LT healing (κ = 0.506).

Anatomical tuberosity healing was observed in 28/40 RSAs (70%), with no significant difference between EFR (15/20, 75%) and non-EFR (13/20, 65%) groups (*p* = 0.490). GT healing was 75% in both groups; LT healing was 85% among patients with available axial views. There were no significant group differences in GT or LT healing, scapular notching, or periarticular ossifications (Table 4).

**Figure 3:**
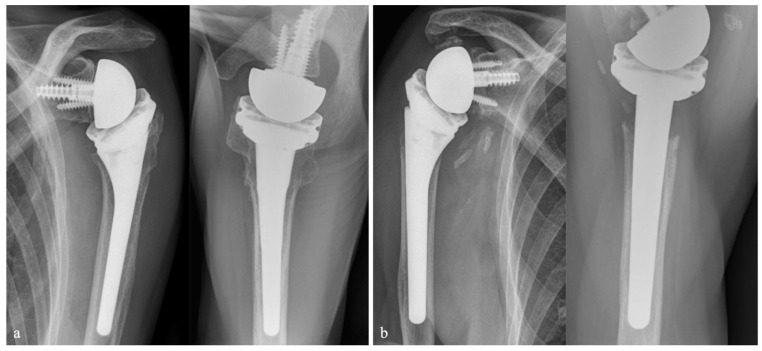
Radiographs at follow-up, (**a**) anatomic GT and LT healing in AP and axial view, (**b**) GT migration with nonunion, ossifications and scapular notching in AP view, GT and LT resorption in axial view (GT: greater tuberosity, LT: lesser tuberosity, AP: anterior–posterior).

Clinical outcomes tended to be better in patients with anatomical tuberosity healing, but no statistically significant associations were observed. Figure 3 shows an example of successful GT healing (Figure 3a) and a case with GT migration, nonunion, tuberosity resorption, ossifications, and scapular notching (Figure 3b).

### 3.4. Adverse Events

One case of deep infection occurred in the EFR group three months postoperatively. It was treated with two-stage revision surgery including partial GT excision. At final follow-up, the patient achieved satisfactory function (ACS 85, SSV 70%, DASH 23).

## 4. Discussion

This retrospective matched-pair analysis compared 40 RSAs for PHFs using EFR versus a conventional protocol with postoperative immobilization (non-EFR). Groups were propensity score matched 1:1 based on relevant patient and surgical factors. EFR demonstrated significantly better CS and ACS, and active external rotation. Tuberosity healing rates were comparable, with GT healing observed in 75% and LT healing in 85% of cases with available imaging. No statistical differences were found in radiographic parameters.

Outcomes after RSA for PHFs are multifactorial and cannot be attributed solely to the rehabilitation protocol. As all patients in this study were treated acutely, variability related to surgical timing was minimized; however, other influencing factors may still have contributed to the observed results.

The overall clinical outcomes and PROMs in our study were consistent with, and in some parameters better than, those previously reported for RSA in PHFs [2,15]. Importantly, the mean CS in the EFR group was 65.5, exceeding the Patient Acceptable Symptom State (PASS) threshold of 52 points for RSA [16]. This is particularly noteworthy given the relatively high mean age of over 80 years in our cohort. Furthermore, the EFR group had significantly higher ASA scores (*p* = 0.046), which is typically associated with inferior clinical outcomes. The difference in CS between the EFR and non-EFR groups was 9.5 points, which was not only statistically significant but also clinically meaningful. The threshold for the Minimal Clinically Important Difference (MCID) in RSA, reported to range between 5.7 and 9.4 points, was exceeded [17,18,19]. In contrast to the MCID, the Substantial Clinical Benefit (SCB), defined as a 19.1 point improvement in CS was not reached in the between-group comparison [20]. However, SCB is typically assessed as a within-group change from pre- to postoperative values and is therefore not directly applicable to between-group comparisons. Considering the clinical relevance thresholds such as MCID and PASS, our findings suggest that EFR results in superior clinical outcomes that are both statistically and clinically meaningful. The improvements observed were not only significant in magnitude but also reached levels of shoulder function that patients perceive as acceptable. These benefits were achieved despite the EFR group having higher comorbidity and more complex fracture patterns, highlighting the potential value of early mobilization after RSA for PHFs.

In addition, the tuberosity healing rates observed in our study are highly promising, with no differences between EFR and non-EFR [6,21]. A systematic review by Jain et al., analyzing seven studies that included conventional postoperative shoulder immobilization for up to six weeks reported an overall GT healing rate of 70.5%, which is consistent with our findings [6]. The integrity of the tuberosities appears to be crucial for achieving good shoulder function, as the authors found that GT healing is associated with improved clinical outcomes. In our study, we observed a similar trend, but no statistically significant differences could be demonstrated. The good clinical outcomes observed in our study are likely associated with the high rate of GT healing. Most studies primarily focus on the healing of the GT, with little attention given to the LT healing [6]. In our study, LT healing was analyzed, but this was limited by the fact that only 65% of the patients had an axial view. Nevertheless, our analysis revealed high LT healing rates, with 85% demonstrating anatomic healing. However, we found no significant correlation between LT healing and clinical outcomes.

The literature comparing EFR and delayed rehabilitation is limited and inconsistent. One recent study by Tuphe et al. found better clinical outcomes with EFR but reported a lower GT healing rate (33%) compared to our rate of 75% [5]. Differences in age, implant design (145° vs. 135° inclination), and surgical technique may explain this. Biomechanical evidence suggests that 135° inclination RSAs with proper tuberosity fixation result in more stable healing [7,20]. Our approach, using horizontal and vertical cerclage sutures and temporary reduction forceps, appears to offer reliable anatomical reconstruction. The low rate of scapular notching (10%) and absence of loosening or instability further support the surgical protocol’s reliability. These findings align with biomechanical and clinical studies favoring 135° RSAs with lateralized components [2,14]. The clinical and radiographic outcomes of our study emphasize that combining this surgical approach with ERF for managing PHFs in older patients represents a promising strategy.

Since no differences were observed in patient or treatment characteristics, nor in tuberosity healing in our study, postoperative rehabilitation protocols appear to play a crucial role in achieving favorable clinical outcomes after RSA. Early rehabilitation may help reduce joint adhesion formation and promote early mobilization, which, in turn, enhances muscular activation, counters muscle atrophy, and accelerates improvements in muscle function. Furthermore, early loading supports better adaptation of soft tissues and periarticular structures to the prosthesis, thereby optimizing overall mobility. Studies consistently demonstrate that EFR yields significantly better outcomes in terms of strength, range of motion, and pain reduction [22]. Studies indicate that EFR may be particularly beneficial for older patients, aiding in the maintenance of independence and improvement of shoulder function [4]. However, there is currently no consensus among surgeons regarding the optimal postoperative protocol for patients undergoing RSA following PHFs [23,24].

This study has several limitations. The small sample size of 40 patients and the retrospective design limit the generalizability of the findings. The lack of randomization in assigning rehabilitation protocols may introduce selection bias although patients in the EFR group had higher ASA scores and more complex fractures which likely biased results against and not in favor of EFR. Propensity score matching reduced confounding but cannot fully eliminate it. Preoperative PROMs were not available due to the acute fracture setting and could therefore not be included in the matching process. Comorbidity was assessed using the ASA classification, while more detailed matching was limited by the small sample size. The higher ASA scores in the EFR group indicate a greater comorbidity burden, which would be expected to bias against better outcomes. The study population consisted of very old and comorbid patients which increases the risk of loss to follow-up. In the EFR group 14 patients could not be included due to death or reduced general health which may have caused attrition bias. Clinical follow-up assessments were not blinded which may have introduced observer bias. Axial radiographs were available in only 65 percent of cases which limited full assessment of lesser tuberosity healing. Furthermore, the assessment of healing may have oversimplified radiographic findings in borderline cases. Tuberosity healing was analyzed by a surgeon and a radiologist. However, the senior author made the final decision regarding the classification of tuberosity healing, which may have introduced additional observer bias. Potential complications were assessed using a standardized approach; however, minor complications may have been underreported due to the retrospective design and the limited follow-up period of 12 to 24 months.

Despite these limitations the matched cohort design, high tuberosity healing rates and clinically relevant improvements in functional outcomes support the robustness of the findings. This study offers valuable insights, particularly regarding the potential benefits of EFR following RSA for PHFs. This is one of the first studies to specifically assess the impact of EFR on both clinical outcomes and tuberosity healing. Our findings provide valuable insights into the potential advantages of EFR protocols for older patients. Based on the clinical and radiological findings of our study, the use of EFR for older patients can be recommended. However, further studies are needed to confirm these results and address the limitations of this study. Randomized controlled trials should be conducted to provide higher-quality evidence on the impact of early functional rehabilitation on both clinical and radiographic outcomes in this population.

## 5. Conclusions

Early functional rehabilitation after reverse shoulder arthroplasty in older adults with proximal humerus fractures was associated with improved functional outcomes without evidence of compromised tuberosity healing. While these findings suggest that early mobilization may be a safe and effective rehabilitation strategy, causal inferences cannot be drawn due to the observational study design. Further prospective randomized studies are warranted to confirm these results.

## Figures and Tables

**Table 1:** Patient characteristics.

Patient Characteristics			
	EFR	Non-EFR	*p*-Value
Age, mean years (SD)	81.5 (6.7)	79.9 (6.4)	^B^ 0.446
Gender, n (%) Female Male	18 (90) 2 (10)	18 (90) 2 (10)	^A^ 1.000
Affected dominant side, n (%)	15 (75)	11 (55)	^A^ 0.185
BMI, mean kg/m^2^ (SD)	24.3 (4.3)	25.8 (4.9)	^B^ 0.297
Smoking, n (%)	4 (20)	3 (15)	^A^ 0.791
Diabetes, n (%)	1 (5)	5 (25)	^A^ 0.077
Rheumatoid arthritis, n (%)	3 (15)	1 (5)	^A^ 0.344
ASA, n (%) ASA type II ASA type III ASA type IV	10 (50) 10 (50) 0 (0)	16 (80) 3 (15) 1 (5)	^A^ 0.046
Neer classification, n (%) 2-part 3-part 4-part	1 (5) 13 (65) 6 (30)	3 (15) 5 (25) 12 (60)	^A^ 0.038
AO classification, n (%) B1 B2 B3 C1 C2 C3	2 (10) 2 (10) 0 (0) 5 (25) 10 (50) 1 (5)	2 (10) 3 (15) 1 (5) 1 (5) 10 (50) 3 (15)	^A^ 0.432
Osteoarthritis, n (%) Samilson–Prieto grade I Samilson–Prieto grade II	11 (55) 3 (15	10 (50) 3 (15)	^A^ 0.940
Follow-up time, median months (IQR)	14.0 (3.5)	17.5 (7.0)	^C^ 0.058

EFR: early functional rehabilitation group, non-EFR: non-early functional rehabilitation group, SD: standard deviation, n: number, IQR: interquartile range, BMI: body mass index, kg: kilogram, m: meter, ASA: American Society of Anaesthesiologists, AO: Arbeitsgemeinschaft für Osteosynthesefragen, ^A^ chi-square, ^B^ *t*-test, ^C^ Mann–Whitney U test.

**Table 2:** Treatment characteristics.

Treatment Characteristics			
	EFR	Non-EFR	*p*-Value
Glenosphere size, n (%) 36 + 4 mm 39 + 4 mm	16 (80) 4 (20)	16 (80) 4 (20)	^A^ 1.000
Baseplate, n (%) Modular glenoid system, 24 mm Universal glenoid, small	17 (85) 3 (15)	17 (85) 3 (15)	^A^ 1.000
Central baseplate fixation, n (%) Central screw Central post	12 (60) 8 (40)	9 (45) 11 (55)	^A^ 0.342

EFR: early functional rehabilitation group, non-EFR: non-early functional rehabilitation group, SD: standard deviation, n: number, mm: millimeter, ^A^ chi-square.

**Table 3:** Patient-reported and clinical outcomes.

Patient-Reported and Clinical Outcomes			
	EFR	Non-EFR	*p*-Value
*Patient-reported outcomes*			
Subjective shoulder value (0–100), mean (SD)	78.0 (13.6)	70.8 (14.4)	^B^ 0.111
VAS pain (0–10), median (IQR)	0 (1.8)	0 (4.0)	^C^ 0.236
Constant score, mean (SD) Pain (0–15), median (IQR) Activities of daily living (0-20), mean (SD) Range of motion (0–40), mean (SD) Strength (0–25), median (IQR)	65.5 (12.5) 15.0 (2.0) 17.5 (2.6) 26.3 (6.1) 6.0 (5.0)	56.0 (15.3) 15.0 (5.3) 15.0 (4.1) 23.1 (8.0) 6.0 (3.3)	^B^ 0.044 ^C^ 0.256 ^B^ 0.026 ^B^ 0.165 ^C^ 0.497
Adjusted Constant score, mean (SD)	72.3 (14.3)	63.5 (15.9)	^B^ 0.030
DASH score, (mean SD)	24.8 (18.4)	34.1 (18.0)	^B^ 0.113
*Active range of motion*			
Flexion, mean ° (SD)	113.9 (27.1)	103.8 (25.3)	^B^ 0.228
Abduction, mean ° (SD)	105.0 (28.0)	97.1 (23.8)	^B^ 0.342
External rotation, mean ° (SD)	25.4 (13.4)	20.7 (15.1)	^B^ 0.002
Internal rotation, n (%) Interscapular T12 vertebra L3 vertebra Lumbosacral junction Buttock Lateral thigh	0 (0) 6 (30) 6 (30) 3 (15) 5 (25) 0 (0)	1 (5) 1 (5) 5 (25) 1 (5) 12 (60) 0 (0)	^A^ 0.074

EFR: early functional rehabilitation group, non-EFR: non-early functional rehabilitation group, SD: standard deviation, n: number, IQR: interquartile range, VAS: visual analogue scale, DASH: Disabilities of the Arm, Shoulder and Hand, ^A^ chi-square, ^B^ *t*-test, ^C^ Mann–Whitney U test.

**Table 4:** Radiographic outcomes.

Radiographic Outcomes				
	EFR	Non-EFR	*p*-Value	Cohen’s Kappa
Greater tuberosity healing, n (%)				
Anatomical healing	15 (75)	15 (75)	^A^ 1.000	0.750
Migration/nonunion Malunion Resorption Partial resorption	3 (15) 1 (5) 0 (0) 1 (5)	3 (15) 2 (10) 0 (0) 0 (0)	^A^ 0.721	0.593
Lesser tuberosity healing, n (%)				
Anatomical healing	11 (85)	11 (85)	^A^ 1.000	0.506
Migration/nonunion Malunion Resorption Partial resorption	0 (0) 0 (0) 1 (8) 1 (8)	1 (8) 0 (0) 0 (0) 1 (8)	^A^ 0.572	0.222
Scapular notching, n (%) None Grade 1 Grade 2	2 (10) 1 (5) 1 (5)	2 (10) 2 (10) 0 (0)	^A^ 0.513	0.691
Ossifications, n (%)	9 (45)	9 (45)	^A^ 1.000	0.650

EFR: early functional rehabilitation group, non-EFR: non-early functional rehabilitation group, n: number, ^A^ chi-square.

## Data Availability

The data used in this study were obtained from the internal documentation of data entry and processing within the German Arthroplasty Registry (Schulterprothesenregister, SPR). Data were used and analyzed in accordance with applicable data protection regulations and data use agreements. Patient consent does not include the publication of raw data; therefore, the complete database is not publicly available.

## Abbreviations

ACS	Age- and gender-adjusted Constant score
AO	Arbeitsgemeinschaft für Osteosynthesefragen
AP	Anteroposterior
ASA	American Society of Anesthesiologists (physical status classification)
BMI	Body mass index
CS	Constant score
DASH	Disabilities of the Arm, Shoulder, and Hand score
EFR	Early functional rehabilitation
GT	Greater tuberosity
IQR	Interquartile ranges
κ	Cohen’s kappa (inter-rater reliability coefficient)
LT	Lesser tuberosity
MCID	Minimal Clinically Important Difference
Non-EFR	Postoperative immobilization protocol without early functional rehabilitation
PASS	Patient Acceptable Symptom State
PHF	Proximal humeral fracture
PROMs	Patient-reported outcome measures
ROM	Range of motion
RSA	Reverse shoulder arthroplasty
SCB	Substantial Clinical Benefit
SD	Standard deviation
SPSS	Statistical Package for the Social Sciences
SSV	Subjective shoulder value
VAS	Visual analogue scale

## Appendix A. Early Functional Rehabilitation Program After Reverse Shoulder Arthroplasty

**Early functional rehabilitation**
Day 1: - Immobilization in the shoulder sling for 24 h. - Cryotherapy. - Active mobilization of the hand and elbow. Week 1 to 6: - Gradual wean off the shoulder sling, based on pain tolerance. - Begin active-assisted exercise therapy without limitations on range of motion. - Initiate active concentric and isometric strengthening exercises for the rotator cuff and deltoid muscles once full flexion is achieved. - Shoulder stabilizer exercises and posture training (e.g., back school). - Gradual increase in weight-bearing, based on pain tolerance. From week 6: - Outpatient or inpatient rehabilitation therapy.

## Appendix B. Non-Early Functional Rehabilitation Program After Reverse Shoulder Arthroplasty

**Non early functional rehabilitation**
Day 1: - Immobilization in the abduction sling for 3 weeks. - Cryotherapy. - Active mobilization of the hand and elbow. Week 1 and 2: - Flexion/abduction up to 60°, initially passive, then also assistive under absolute pain-free conditions. Mobilize from internal rotation (IRO) to 0° active rotation (ARO). - Active mobilization of the elbow with the arm adducted, incorporating dynamic, pain-free biceps and triceps exercises. Week 3 and 4: - Active flexion up to 90°, without lifting. Active abduction up to 60°, without lifting. Passive internal rotation (IRO) up to 80° before the body’s longitudinal axis, pain-free. - Concentric-eccentric training of the scapular stabilizers. Week 5 and 6: - Progressing to full flexion - Active abduction up to 90°, internal rotation (IRO) up to 80° active and pain-free, continue limiting active external rotation (ARO) to 0°. From week 6: - Outpatient or inpatient rehabilitation therapy. - Working towards full abduction - Start mobilizing external rotation (ARO). - Training of the rotator cuff, deltoid, and shoulder stabilizers.

## References

[1] Fraser A.N., Bjørdal J., Wagle T.M., Karlberg A.C., Lien O.A., Eilertsen L., Mader K., Apold H., Larsen L.B., Madsen J.E. (2020). Reverse Shoulder Arthroplasty Is Superior to Plate Fixation at 2 Years for Displaced Proximal Humeral Fractures in the Elderly: A Multicenter Randomized Controlled Trial. J. Bone Jt. Surg..

[2] Minarro J.C., Sanchez-Sotelo J. (2024). Reverse Shoulder Arthroplasty for Proximal Humerus Fractures: A Review of Current Evidence. Curr. Rev. Musculoskelet. Med..

[3] Kuechly H.A., Perry A.K., Grawe B.M. (2024). Reverse shoulder replacement for the treatment of proximal humerus fractures: A current literature review. JSES Int..

[4] Sabesan V.J., Gilot G., Chatha K., Grunhut J., Brown S., Lavin A.C. (2022). The effect of an early mobilization rehabilitation protocol on outcomes after reverse shoulder arthroplasty. Semin. Arthroplast. JSES.

[5] Tuphe P., Caubriere M., Hubert L., Lancigu R., Sakek F., Loisel F., Obert L., Rony L. (2023). Early rehabilitation after reverse total shoulder prosthesis on fracture of proximal humerus in elderly patients provides better functional outcome. Eur. J. Orthop. Surg. Traumatol..

[6] Jain N.P., Mannan S.S., Dharmarajan R., Rangan A. (2019). Tuberosity healing after reverse shoulder arthroplasty for complex proximal humeral fractures in elderly patients—Does it improve outcomes? A systematic review and meta-analysis. J. Shoulder Elb. Surg..

[7] Schmalzl J., Piepenbrink M., Buchner J., Picht S., Gerhardt C., Lehmann L.-J. (2021). Higher primary stability of tuberosity fixation in reverse fracture arthroplasty with 135° than with 155° humeral inclination. J. Shoulder Elb. Surg..

[8] Gilbart M.K., Gerber C. (2007). Comparison of the subjective shoulder value and the Constant score. J. Shoulder Elb. Surg..

[9] Constant C.R., Murley A.H. (1987). A clinical method of functional assessment of the shoulder. Clin. Orthop. Relat. Res..

[10] Tavakkolizadeh A., Ghassemi A., Colegate-Stone T., Latif A., Sinha J. (2009). Gender-specific Constant score correction for age. Knee Surg. Sports Traumatol. Arthrosc..

[11] Gummesson C., Atroshi I., Ekdahl C. (2003). The disabilities of the arm, shoulder and hand (DASH) outcome questionnaire: Longitudinal construct validity and measuring self-rated health change after surgery. BMC Musculoskelet. Disord..

[12] Neer C.S. (1970). Displaced proximal humeral fractures. I. Classification and evaluation. J. Bone Jt. Surg. Am..

[13] Müller M.E., Koch P., Nazarian S., Schatzker J. (1990). Humerus = 1. The Comprehensive Classification of Fractures of Long Bones.

[14] Sirveaux F., Favard L., Oudet D., Huquet D., Walch G., Mole D. (2004). Grammont inverted total shoulder arthroplasty in the treatment of glenohumeral osteoarthritis with massive rupture of the cuff: Results of a multicentre study of 80 shoulders. J. Bone Jt. Surg. Br. Vol..

[15] Lu V., Jegatheesan V., Patel D., Domos P. (2023). Outcomes of acute vs. delayed reverse shoulder arthroplasty for proximal humerus fractures in the elderly: A systematic review and meta-analysis. J. Shoulder Elb. Surg..

[16] Yang C., Yang A.Z., Xu S., Yew A., Lie D.T.T. (2024). Determining patient acceptable symptom states from patient reported outcome measures following reverse shoulder arthroplasty: Constant-murley, UCLA, Oxford Shoulder Scores. J. Orthop..

[17] Simovitch R.W., Elwell J., Colasanti C.A., Hao K.A., Friedman R.J., Flurin P.H., Wright T.W., Schoch B.S., Roche C.P., Zuckerman J.D. (2024). Stratification of the minimal clinically important difference, substantial clinical benefit, and patient acceptable symptomatic state after total shoulder arthroplasty by implant type, preoperative diagnosis, and sex. J. Shoulder Elb. Surg..

[18] Torrens C., Corrales M., Vilà G., Santana F., Cáceres E. (2011). Functional and Quality-of-Life Results of Displaced and Nondisplaced Proximal Humeral Fractures Treated Conservatively. J. Orthop. Trauma.

[19] Dabija D.I., Jain N.B. (2019). Minimal Clinically Important Difference of Shoulder Outcome Measures and Diagnoses: A Systematic Review. Am. J. Phys. Med. Rehabil..

[20] Simovitch R., Flurin P.-H., Wright T., Zuckerman J.D., Roche C.P. (2018). Quantifying success after total shoulder arthroplasty: The substantial clinical benefit. J. Shoulder Elb. Surg..

[21] O’Sullivan J., Lädermann A., Parsons B.O., Werner B., Steinbeck J., Tokish J.M., Denard P.J. (2020). A systematic review of tuberosity healing and outcomes following reverse shoulder arthroplasty for fracture according to humeral inclination of the prosthesis. J. Shoulder Elb. Surg..

[22] Nolan L., Mahon J., Mirdad R., Alnajjar R., Galbraith A., Kaar K. (2024). Early mobilization versus immobilization after reverse shoulder arthroplasty and total shoulder arthroplasty: A systematic review. Bone Jt. J..

[23] Patch D.A., Reed L.A., Hao K.A., King J.J., Kaar S.G., Horneff J.G., Ahn J., Strelzow J.A., Hebert-Davies J., Little M.T. (2022). Understanding postoperative rehabilitation preferences in operatively managed proximal humerus fractures: Do trauma and shoulder surgeons differ?. J. Shoulder Elb. Surg..

[24] Tong C.H., Fang C.X. (2023). Rehabilitation progress following reverse total shoulder replacement and internal fixation for geriatric three and four-part proximal humerus fractures—A propensity score matched comparison. BMC Musculoskelet. Disord..

